# About the future of GMS German Medical Science

**DOI:** 10.3205/000364

**Published:** 2026-08-10

**Authors:** Manfred Gogol

**Affiliations:** 1 Trauma Department, Hannover Medical School, Hannover, Germany

## About the future of GMS German Medical Science

As I became the editor-in-chief several years ago, GMS German Medical Science was a somewhat well-known as well as under-recognized open access journal. The quality of submissions varied, but over time the development of the journal was slow but improving. Thus, as of July 20, 2026, Scopus CiteScore notices GMS German Medical Science with a score of 3.3 [1]. As of July 5, 2026, CiteScoreTracker reports a citation rate of 1.9 and the Source Normalized Impact per Paper (SNIP) is 1.134. The SCImago index of the journal is 0.344 for 2026 [2]. The citations assessed by SCImago regarding the last three years are shown in Table 1 (Tab. 1).

We can assume that corresponding to these numbers the Journal Impact Factor (JIF) produced by Clarivate will be approximately 2. Following discussions with ZB MED – Information Centre for Life Sciences (https://www.zbmed.de/en/), where GMS German Medical Science is produced and hosted, and with the board of the Association of Scientific Medical Societies in Germany, a few changes will be implemented in the future.

### Introducing publication fees

Due to public funding in the past years, the journal GMS German Medical Science did not demand publication fees from authors of accepted papers or their institutions. But within the last years, especially in 2026, public funding has become more and more restricted and in future the production of the journal cannot be offered for free any longer. Therefore, we have decided to introduce an article fee of € 500 ($ 570, £ 425) plus VAT, which is 19% in Germany. This change will become effective for submissions on September 1, 2026, and later. The fee does not cover the production cost and the hosting service, but it will in part compensate the restricted public funding for ZB MED. These fees are clearly lower than the value of published articles and the cost of production and hosting are today. Please notice that SCImago judged the article processing charges for GMS German Medical Science to be approximately $ 3,000 [2]. The Association of Scientific Medical Societies in Germany, which runs this journal, thus makes no profit and commits itself to following the principles of FAIR publishing.

### Using artificial intelligence

The development of artificial intelligence (AI) within the last years in scientific research and publishing makes it necessary to establish guidelines, especially for journals. So, in future authors will have to state clearly if and for what aspects of their submission AI was used [3].

### Updating GMS German Medical Science in general

Within the next weeks and months the journal GMS German Medical Science will update its aims and scope as well as the editorial board (https://journals.publisso.de/en/journals/gms/about). Additionally, one major task will be to refresh and enlarge our reviewer community to be able to provide high quality reviews for submissions in a timely manner. So, if you are interested in becoming a reviewer or if you are a senior clinician and researcher and know someone for whom the task might be helpful regarding the development of their skills, please contact us.

### Applying for Web of Science indexing (Journal Impact Factor)

In general, GMS German Medical Science as well as the Association of Scientific Medical Societies in Germany are critical about the JIF, because it is often misused. Nevertheless, the JIF is the major currency in science. The journal has been recognized in MEDLINE and PubMed Central for years and access to and downloads of published papers display an increasing recognition in the scientific community. To foster this process, we have decided to apply for indexing in the Web of Science, which includes the assignment of a JIF, within this year.

### Outlook

Overall, we believe in research, integrity in research and publishing, and publishing open access. The experience of the last years makes us optimistic that changing times and conditions will not weaken but strengthen GMS German Medical Science. For this purposes the scientific society is invited to participate in the future process and the development of the journal.

## Notes

### Competing interests

The author declares that he has no competing interests.

## Figures and Tables

**Table 1:**
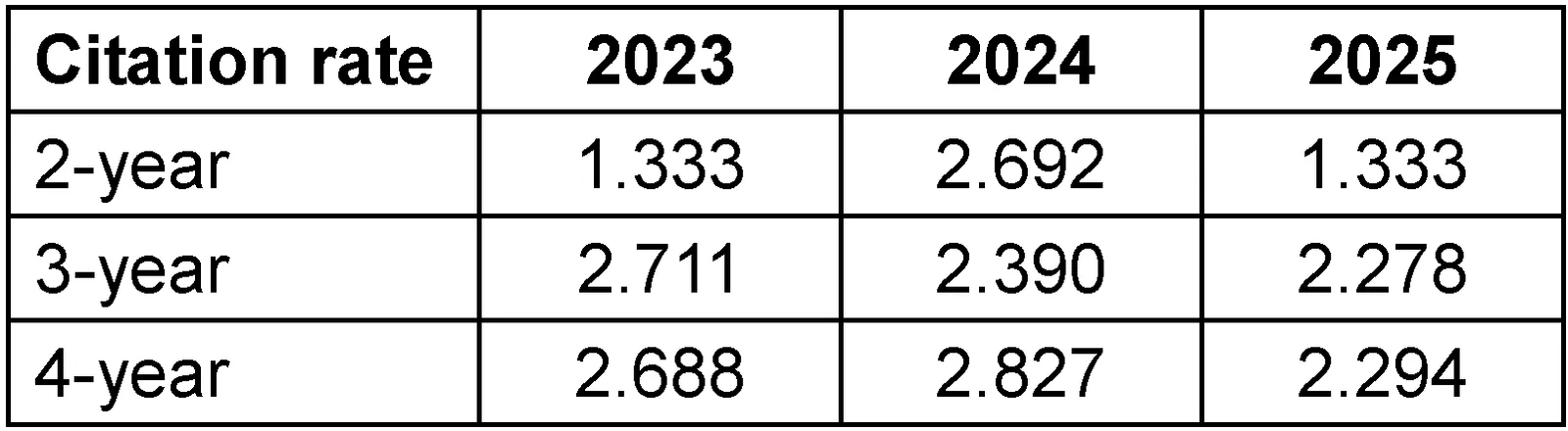
Citation rate 2023–2025 by SCImago [2]
